# Supplementary material for: Predicting visual function by interpreting a neuronal wiring diagram
Source: Nature. 2024 Oct 2;634(8032):113–23. doi: 10.1038/s41586-024-07953-5 (PMC11446822; doi:10.1038/s41586-024-07953-5)
Supplement: Supplementary file 6 — CRF and ERF predictions for individual TmY4 and TmY9 cells. Analogous to Supplementary Data 3, but for TmY target types. Shown are the top four monosynaptic pathways, the strongest pathway passing through each of the top ten intermediary types (ranking from Extended Data Fig. 7), and the trisynaptic pathway Tm1–TmY–Dm3–TmY (see the section entitled Prediction of spatial normalization). [file 41586_2024_7953_MOESM6_ESM.zip › DataS4/TmY9q/720575940626583838.pdf]

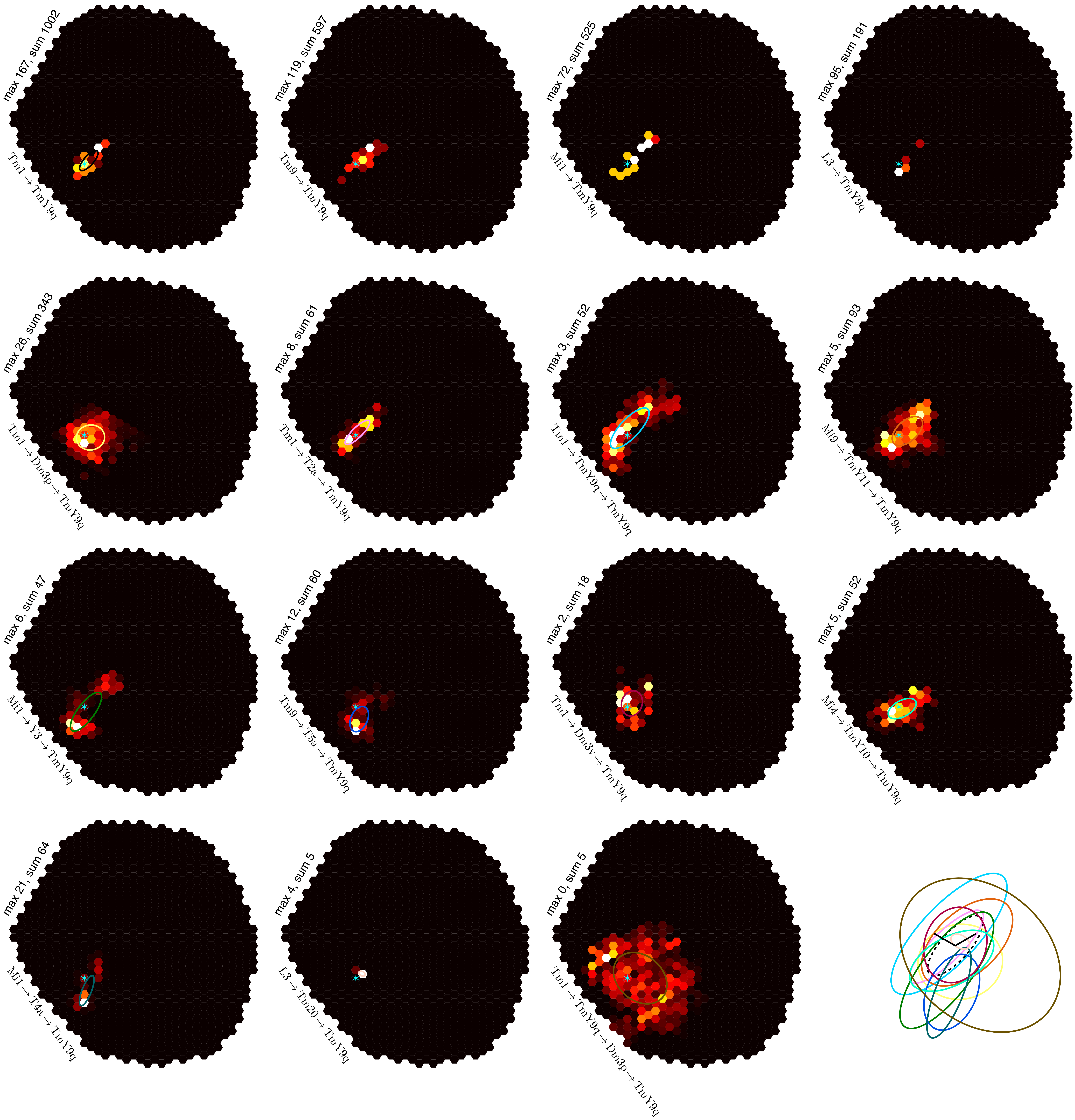
max 167, sum 1002
Tm1 → TmY9q
max 119, sum 597
Tm9 → TmY9q
max 72, sum 525
Mi1 → TmY9q
max 95, sum 191
L3 → TmY9q
max 26, sum 343
Tm1 → Dm3p → TmY9q
max 8, sum 61
Tm1 → T2a → TmY9q
max 3, sum 52
Tm1 → TmY9q → TmY9q
max 5, sum 93
Mi9 → TmY11 → TmY9q
max 6, sum 47
Mi1 → Y3 → TmY9q
max 12, sum 60
Tm9 → T5a → TmY9q
max 2, sum 18
Tm1 → Dm3v → TmY9q
max 5, sum 52
Mi4 → TmY10 → TmY9q
max 21, sum 64
Mi1 → T4a → TmY9q
max 4, sum 5
L3 → Tm20 → TmY9q
max 0, sum 5
Tm1 → TmY9q → Dm3p → TmY9q
